# αB-crystallin is a sensor for assembly intermediates and for the subunit topology of desmin intermediate filaments

**DOI:** 10.1007/s12192-017-0788-7

**Published:** 2017-05-03

**Authors:** Sarika Sharma, Gloria M. Conover, Jayne L. Elliott, Ming Der Perng, Harald Herrmann, Roy A. Quinlan

**Affiliations:** 1 Division of Molecular Genetics, German Cancer Research Center, Heidelberg, Germany; 2 Department of Biochemistry and Biophysics, Texas A&M University, College Station, TX USA; 3 Department of Biosciences and the Biophysical Sciences Institute, University of Durham, Durham, UK; 4 Institute of Molecular Medicine, College of Life Sciences, National Tsing Hua University, Hsinchu, 300 Taiwan; 5 Institute of Neuropathology, University Hospital Erlangen, Erlangen, Germany

**Keywords:** Chaperone, CRYAB, Intermediate filaments, Desmin, Desminopathy, Cardiomyopathy

## Abstract

Mutations in the small heat shock protein chaperone CRYAB (αB-crystallin/HSPB5) and the intermediate filament protein desmin, phenocopy each other causing cardiomyopathies. Whilst the binding sites for desmin on CRYAB have been determined, desmin epitopes responsible for CRYAB binding and also the parameters that determine CRYAB binding to desmin filaments are unknown. Using a combination of co-sedimentation centrifugation, viscometric assays and electron microscopy of negatively stained filaments to analyse the in vitro assembly of desmin filaments, we show that the binding of CRYAB to desmin is subject to its assembly status, to the subunit organization within filaments formed and to the integrity of the C-terminal tail domain of desmin. Our in vitro studies using a rapid assembly protocol, C-terminally truncated desmin and two disease-causing mutants (I451M and R454W) suggest that CRYAB is a sensor for the surface topology of the desmin filament. Our data also suggest that CRYAB performs an assembly chaperone role because the assembling filaments have different CRYAB-binding properties during the maturation process. We suggest that the capability of CRYAB to distinguish between filaments with different surface topologies due either to mutation (R454W) or assembly protocol is important to understanding the pathomechanism(s) of desmin-CRYAB myopathies.

## Introduction

The α-crystallins are small heat shock proteins (sHSPs); (Carra et al. 2013; Kappe et al. 2003) that are part of the molecular chaperone family (Brandvold and Morimoto 2015; Kim et al. 2013; Treweek et al. 2014). Protein chaperones assist the folding of nascent protein chains, bind to and prevent the aggregation of misfolded and stress-denatured proteins (Brandvold and Morimoto 2015; Strauch and Haslbeck 2016), as well as assisting oligomer assembly and the assembly of protein complexes (Ellis 2013). Their role as assembly chaperones is often overshadowed by their folding pathway(s) function (Ellis 2013). For sHSPs and particularly for αB-crystallin (CRYAB, HSPB5), its role as an assembly chaperone is critical to its many functions in the cell that involve multiprotein complexes (Quinlan and Ellis 2013). Mutations in sHSPs cause many different diseases (Carra et al. 2013; Treweek et al. 2014) typified in their histopathology by protein aggregates of intermediate filaments (IFs) and associated proteins (Perng et al. 2016; Quinlan and Van Den Ijssel 1999). The α-crystallin complex (CRYAA and CRYAB) in the eye lens was first found to modulate the assembly of lenticular IFs (Nicholl and Quinlan 1994). Subsequently, mutations in both CRYAB (Vicart et al. 1998) and the muscle-specific IF protein desmin (Goldfarb et al. 1998) were found to phenocopy each other, causing cardiomyopathy. This first disease-causing mutation in CRYAB, R120G (Vicart et al. 1998), decreased the dissociation constant by half for desmin, causing the assembled desmin filaments to aggregate in both in vitro assembly assays and in transfected cells (Perng et al. 1999b; Perng et al. 2004). Cytosolic heterogeneous multiprotein aggregation and mitochondrial abnormalities characterize myopathies caused by human mutations in desmin or CRYAB (reviewed in (Capetanaki et al. 2015; Kley et al. 2016). Thus, the phenocopying by each highlights the importance of exploring in more detail the desmin-CRYAB interaction.

Desmin is part of a large multigene family of IF cytoskeletal proteins responsible for the mechanical and stress-coping resilience of cells (Guo et al. 2013) and no more so than in muscle (Palmisano et al. 2014) where the targeted deletion of desmin from the mouse genome causes cardiomyopathy, fibrosis and heart failure (Milner et al. 1996). The desmin network is recognized as a key mechanical element (Kiss et al. 2006; Li et al. 2013; Li et al. 1997; Palmisano et al. 2015; Wojtowicz et al. 2015) that can become progressively dysfunctional through myopathy-causing desmin mutations (Kreplak and Bar 2009).

Desminopathy is a rare neuromuscular disorder, belonging to the so-called myofibrillar myopathies, caused by inherited mutations in *DES* (Clemen et al. 2013; Palmio and Udd 2016). A major histopathological feature of desminopathy is the accumulation of insoluble desmin and partner proteins including CRYAB into aggregates (Maerkens et al. 2013). The optimal biomechanical properties for muscle sarcomeres depend heavily upon desmin (Conover et al. 2009; Diokmetzidou et al. 2016; Li et al. 2013; Palmisano et al. 2014) and CRYAB (Diokmetzidou et al. 2016; Wojtowicz et al. 2015). Thus far, it is only the desmin-binding sites on CRYAB that are known (Houck et al. 2011), but those domains in desmin that determine CRYAB binding are unknown. According to pin array studies, three surface exposed peptides within two beta strands of CRYAB and a C-terminal peptide had the strongest binding sites to desmin in a temperature-dependent manner (Ghosh et al. 2007).

The direct interaction of sHSPs and IFs has been proposed to have important cytoprotective roles during normal muscle physiology (Capetanaki et al. 2015; Wettstein et al. 2012). Deeper molecular insights are needed to understand how the assembly of desmin filaments and its network is impacted by CRYAB. There is precedence for desmin-binding partners to affect desmin assembly as recent studies show a delay of a mutant desmin assembly occurs when it binds to nebulin, a giant actin-binding skeletal muscle protein (Baker et al. 2013), or to nebulette, an actin-binding cardiac muscle protein (Hernandez et al. 2016). These studies help explain the molecular basis for the pathology found in desminopathy patients that carry the filament-forming mutant desmin E245D. Furthermore, it is known that other disease-associated desmin mutations alter filament morphology, whilst others dramatically halt filament assembly at distinct stages (Bar et al. 2007b; Bar et al. 2005; Bar et al. 2010). As a major chaperone in muscle (Kato et al. 1991), CRYAB has been suggested to modulate filament assembly and network formation (Perng et al. 1999b; Perng and Quinlan 2015; Perng et al. 2004). CRYAB is a major component in the desmin aggregates taken from muscle biopsies of patients with desminopathies (Maerkens et al. 2013). Thus far, there are no data to suggest that filament morphology and subunit topology affect CRYAB binding. Here, we have investigated the chaperone function of CRYAB in desmin assembly in vitro, using different buffer conditions to manipulate the assembly pathway and alter filament morphology (Herrmann et al. 1999; Herrmann et al. 1996) and find that this influences CRYAB to desmin filaments. We show that the non-α-helical C-terminal domain (“tail”) of desmin contributes to CRYAB binding, and we provide evidence that the mutations I451M and R454W, which map to this domain, alter CRYAB binding. Our data support a sensor role for CRYAB (McHaourab et al. 2009) with respect to desmin filaments and their surface topology.

## Materials and methods

### Cloning, mutagenesis and recombinant protein expression

Point mutations were introduced into the full-length clone of human desmin cDNA by site-directed mutagenesis (Quickchange, Stratagene) using the listed primers (supplemental materials). For protein expression, cDNAs of WT desmin, point mutations and deletion constructs were subcloned into the T5 promoter-driven prokaryotic expression vector pDS5, modified to contain a proper Shine-Dalgarno sequence followed by a *Nco*I restriction site CCATGG, the ATG sequence of which was used as translation start (Herrmann et al. 1993). The identity of the clones was verified by sequencing. Plasmids were expressed in *Escherichia coli* strains TG1 (Amersham Biosciences) or JM109 (Novagen) for protein purification from inclusion bodies followed by cation and anion exchange chromatography, as described (Herrmann et al. 1999). Protein size and purity was verified by SDS PAGE and colloidal Coomassie Brilliant Blue G-250 (CBB; data not shown) staining of the gel. Human recombinant CRYAB was purified as described (Perng et al. 1999a).

### Protein dialysis, desmin assembly, co-sedimentation and viscometry assays

Desmin filament morphology was changed using three different assembly buffers taken from previously published protocols (Herrmann et al. 1996; Mucke et al. 2004; Perng et al. 1999a). Very low ionic strength buffers (preassembly buffers, see below) maintained desmin in a soluble form, such as tetramers or their pre-unit-length filament oligomers (Mucke et al. 2004), prior to initiating assembly by the addition of an equal volume of “assembly buffer” (“Phosphate-buffer”—2 mM sodium phosphate 100 mM NaCl pH 7.5 (Mucke et al. 2004); “Tris-buffer”—100 mM NaCl, 40 mM Tris-HCl, pH 7.0 (Herrmann et al. 1996); “Imidazole-buffer”—200 mM imidazole-HCl, 2 mM DTT (Perng et al. 1999a)) at the indicated temperatures and times. Corresponding low ionic strength preassembly buffers were “low-phosphate”—2 mM Na2PO4, 1 mM DTT pH 7.5; “low-Tris” —5 mM Tris-HCl, 1 mM EDTA, 0.1 mM EGTA, 1 mM DTT, pH 8.4; “low-imidazole”—10 mM Tris-HCl, pH 8.0, 1 mM DTT, 0.2 mM PMSF. The final pH after mixing of equal volumes of preassembly and assembly buffers for the three different buffer compositions was pH 7.5. To monitor the binding of CRYAB to desmin filaments, equimolar ratios of CRYAB and desmin (0.3 μg/μl desmin and 0.12 μg/μl CRYAB) were mixed together in the corresponding low salt buffer prior to adding the assembly buffer. CRYAB was directly dialyzed into the corresponding low salt buffer used to investigate desmin binding.

Buffer pH influences the staging of IF assembly in a tetramer-dependent fashion (Mucke et al. 2004; Wickert et al. 2005). For desmin, reducing the pH from 8.4 to 7.5 arrests the assembly process at an intermediate stage between tetramer complex and unit-length filament (ULF; (Wickert et al. 2005)). Samples were dialyzed overnight into “low-Tris” preassembly buffers at two different pHs: 5 mM Tris-HCl, 1 mM EDTA, 0.1 mM EGTA, 1 mM DTT, pH 8.4 or pH 7.4. When mixed with an equal volume of assembly-inducing “Tris-buffer”, the final pHs of the assembly mixture were 7.5 and 7.1, respectively. To optimise CRYAB binding to desmin still further, the following preassembly buffers, all adjusted to pH 7.4, were tested: “5.0 mM low Tris” (5.0 mM Tris-HCl, 1 mM EDTA, 0.1 mM EGTA, 1 mM DTT), “2.5 mM low Tris” (2.5 mM Tris-HCl, 0.5 mM EDTA, 0.05 mM EGTA, 1 mM DTT) and “1.0 mM low Tris” (1.0 mM Tris-HCl, 0.2 mM EDTA, 0.02 mM EGTA, 1 mM DTT). Assembly was performed at 37 °C for 1 h by adding an equal volume of the assembly “Tris-buffer”, leading to a final pH of 7.1 and final Tris-HCl concentrations of 22.50, 21.25 and 20.50 mM, respectively.

Samples were centrifuged at 30,000 rpm for 30 min at 20 °C in a Beckman centrifuge with swing-out TLS 55 rotor (Beckman Coulter). Viscometry measurements (Ostwald viscometer; Cannon-Nanning) were made as described (Schopferer et al. 2009). For diameter measurements, protein samples were dialyzed and assembled the same day. Data are reported as mean ± SD. A two-sided Student’s *t* test is used to determine the significant differences (*p* value <0.05).

### Band densitometry quantification

Individual protein bands from colloidal CBB-stained and subsequently scanned gels were quantified with ImageJ (http://rsb.info.nih.gov/ij), and the values obtained for each fraction were graphed using Prism 5.0. Experiments were performed in triplicate to make statistical evaluations.

### Electron microscopy

Proteins were fixed in 0.2% (*v*/*v*) glutaraldehyde and mounted on glow-discharged formvar carbon-coated 200-mesh copper grids (SPI Supplies, USA) or on self-coated grids. Samples were negatively stained with 0.2% (*w*/*v*) uranyl acetate for 20 s, and images were acquired on a CCD camera in a Zeiss 900 transmission electron microscope (Carl Zeiss, Germany) at various magnifications between 21,000× and 112,000× at 80 kV. Images were processed for presentation using Adobe CS6. For the measurement of filament diameters, EM images were processed using ImageJ 1.32j. At least 100 measurements were carried out per sample, the mean and SEM calculated, significance determined using Student’s *t* test. All reported *p* values were two-sided and considered to be statistically significant at *p* < 0.05.

## Results

### The morphology of the desmin filaments influences CRYAB binding

**Fig. 1:**
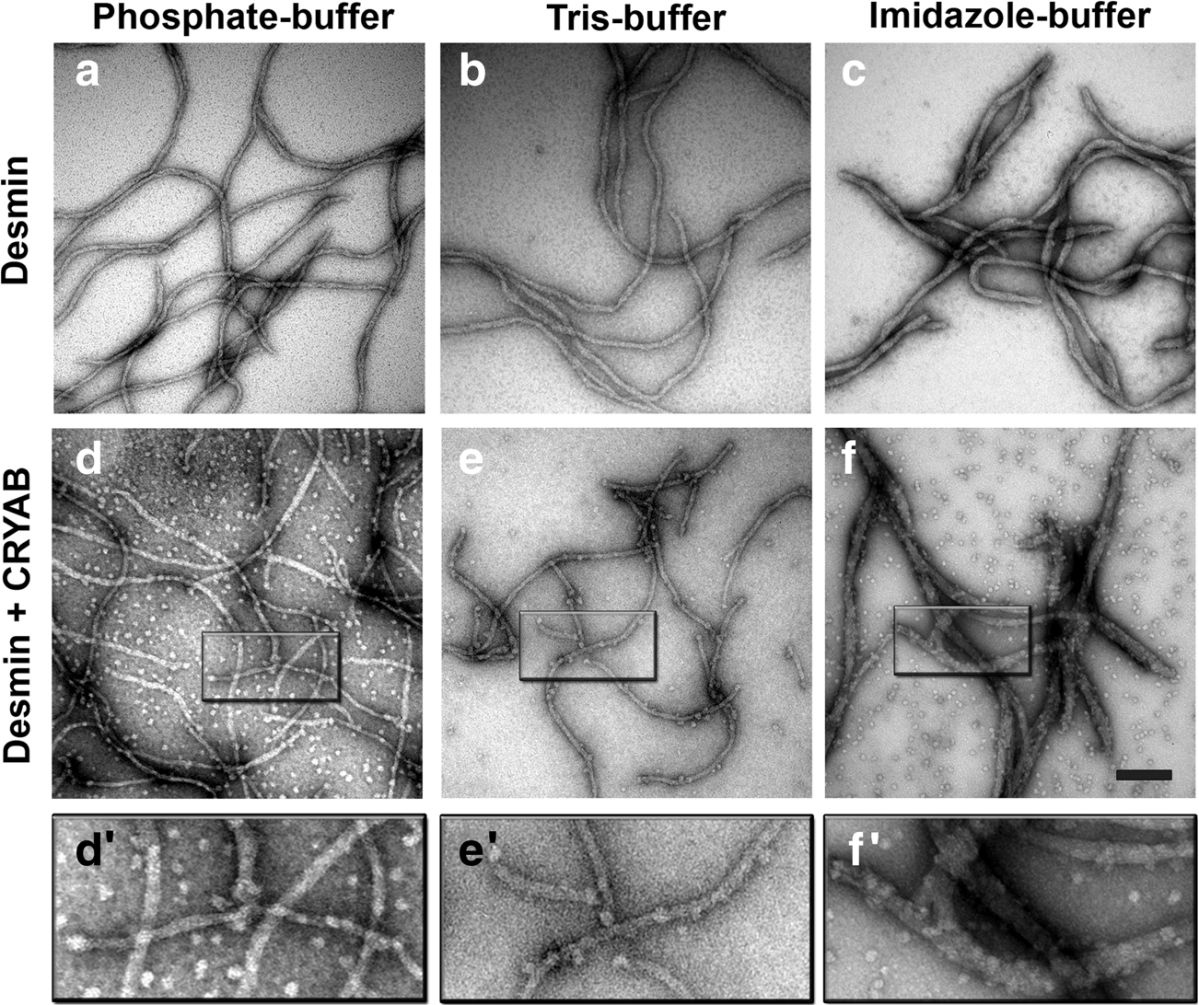
Buffer-dependent desmin filament morphology influences association of CRYAB. The filament structure of negatively stained desmin samples in the presence and absence of CRYAB was compared in “Phosphate-buffer” (**a**, **d**), “Tris-buffer” (**b**, **e**) and “Imidazole-buffer” (**c**, **f**) systems. Assembly reactions were stopped at 60 min by addition of 0.1% (*v*/*v*) glutaraldehyde prior to electron microscopy. The desmin intermediate filament width was preserved in “Phosphate-buffer” (11.5 nm) and “Tris-buffer” (12.3 nm), but was significantly wider in “Imidazole-buffer” (25.8 nm). In the Tris buffer, CRYAB bound along short desmin filaments in a regular manner (**e**), whilst less binding was observed for phosphate buffer and imidazole buffer (**d**’, **f**’). *Insets* show the position of CRYAB oligomers aligned along desmin filaments (see arrows, **d**’–**f**’). Scale bar = 100 nm

The assembly of desmin is achieved in vitro by the removal of chaotropes, the reduction in pH to physiological values (7.0–7.5) and the provision of cations (Herrmann and Aebi 2016). Different assembly protocols have been developed to generate filaments that vary in width and length (Herrmann and Aebi 2016; Herrmann et al. 1999; Wickert et al. 2005). When these protocols were followed, then desmin filament morphology was changed (Fig. 1). In “Phosphate-buffer”, desmin assembled into filaments with continuously smooth extended filament networks (Fig. 1a) as also seen for “Tris-buffer” (Fig. 1b), whereas in the “Imidazole-buffer” (Fig. 1c), desmin filaments were thicker (~25 nm), noncontinuous and tapered particularly towards the filament ends. In “Tris-buffer”, filament diameters were measured as 12.3 ± 1.6 nm, 11.5 ± 1.6 in “Phosphate-buffer” and 25.8 ± 4.3 nm in “Imidazole-buffer”. Filament diameter is proportional to the number of molecules per cross section of the filament (Bar et al. 2004; Herrmann et al. 1999). These data indicate that the assembly buffer and the assembly protocol can significantly influence desmin filament morphology and therefore providing an in vitro strategy to see how filament morphology affects CRYAB binding.

**Fig. 2:**
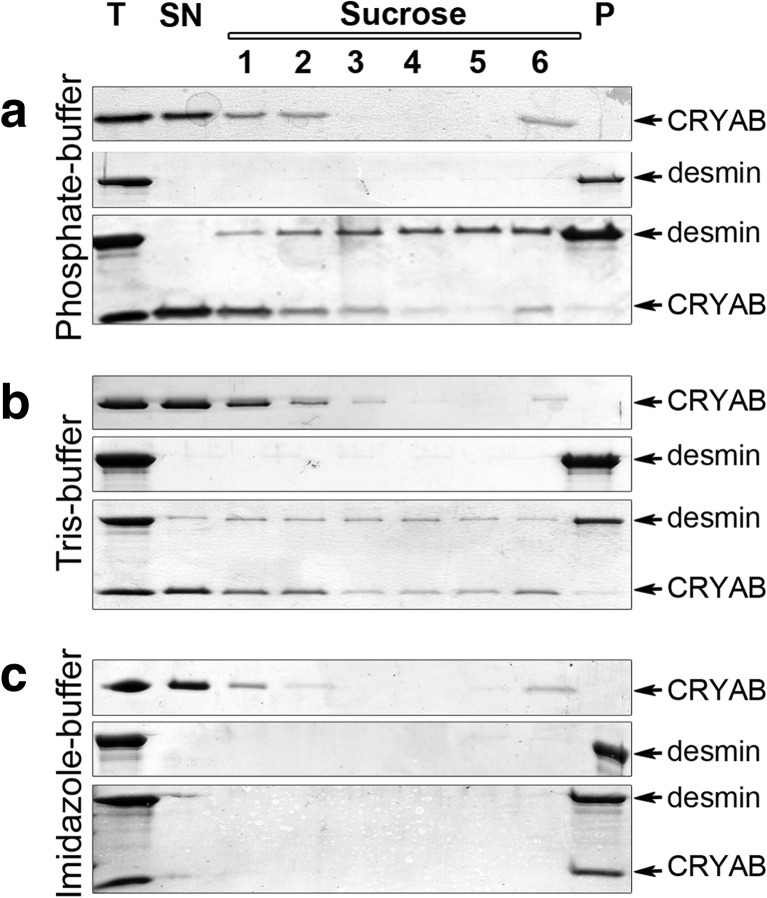
Assembly buffers influence CRYAB binding to desmin filaments**.** The binding of WT desmin to CRYAB was assessed in **a**) “Phosphate-buffer”, (**b**) “Tris-buffer” and (**c**) “Imidazole-buffer” by co-sedimentation assay. CRYAB is present in the supernatant and is also present in the sucrose fractions (1, 2, 6) where desmin is also found. Desmin filaments were found in the insoluble pellet fractions for all three buffer systems. Both proteins were detected in all of the sucrose fractions and were partially found in the pellet in the “phosphate-buffer” and “Tris-buffer”. In contrast, CRYAB was only recovered together with desmin in the insoluble pellet fraction in the imidazole buffer system

Compared to the other two buffers (Fig. 1d, e’), the “Imidazole-buffer” assembled desmin filaments were obviously extensively decorated with CRYAB particles (Fig. 1f, f’). Biochemical co-sedimentation assays revealed comparable binding in “Tris-buffer” and “Phosphate-buffer”, but with noticeably increased levels of desmin in the soluble pools (Fig. 2). This observation is consistent with previous studies reporting high levels of soluble pools of vimentin and GFAP proteins when bound to CRYAB (Nicholl and Quinlan 1994). In agreement with the EM data, the highest levels of desmin and CRYAB in the pellet fractions were found for the “Imidazole-buffer” (Fig. 2c), and these are the filaments that are morphologically the most distinct of the three assembly regimes we investigated.

### Optimisation of the binding of CRYAB to desmin.

**Fig. 3:**
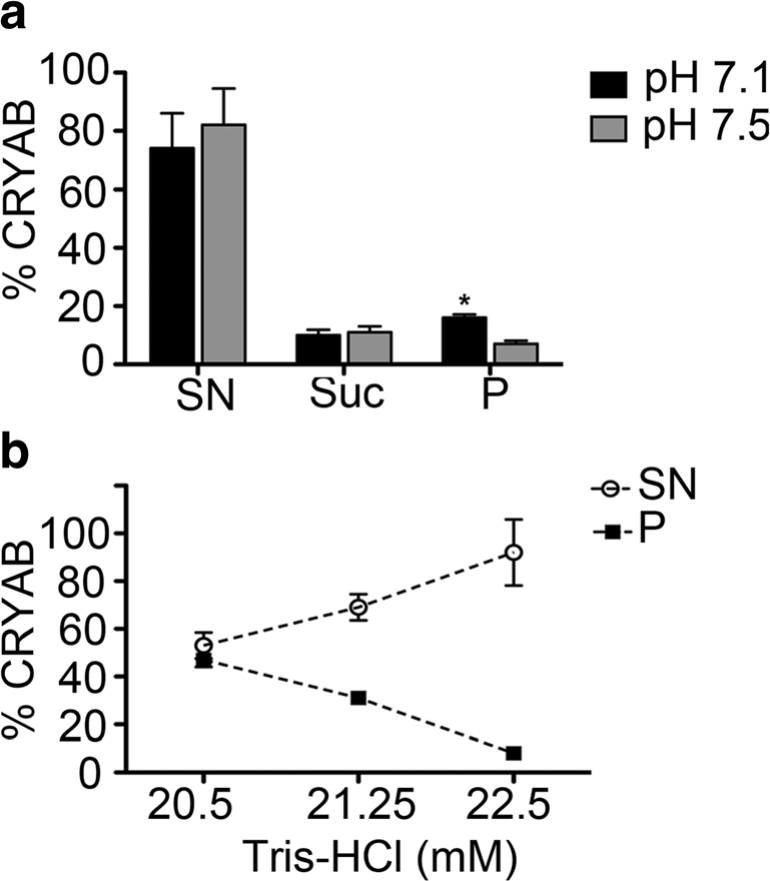
Optimization of CRYAB binding to desmin filaments. The band intensities of co-sedimentation fractions (*SN* supernatant, *Su* sucrose, *P* pellet) from CBB-stained gels were determined. The mean ± SD of three independent experiments is shown. **a** At a constant ionic strength, but varying pH, the binding of CRYAB to desmin filaments increased from 7% (pH 7.5) to 16% (pH 7.1). **b** At pH 7.1, the binding of CRYAB to desmin filaments could be increased from 8% (22.5 mM Tris-HCl) to 31% (21.25 mM Tris-HCl) and finally to 47% (20.5 mM Tris-HCl) by altering the Tris concentration of the “low-Tris” preassembly buffer. Note that the proportion of CRYAB decreases in the supernatant, but increases in the pellet

To biochemically characterize the association of CRYAB with desmin during filament assembly, it was necessary to optimize the Tris buffer conditions to study their in vitro binding properties. To this end, the pH and Tris concentration of the “low-Tris” buffers used at the preassembly stage of the assembly were varied in two separate experiments (Fig. 3a, b).

Our data consistently show that more CRYAB (~16%) was recovered when a final pH of 7.1 was realized rather than pH 7.5, (~7%; Fig. 3a) in “Tris-buffer”. EM analyses indicated that the desmin filament diameter was similar when the assembly reaction was completed in pH 7.1 and 7.5 by the addition of an equal volume of assembly “Tris-buffer” (data not shown). Additionally, our data show that CRYAB binding to desmin could be enhanced further by reducing the Tris concentration of the “low-Tris” buffer from 5 to 1 mM (Fig. 3b), whilst maintaining the pH of the assembly conditions at pH 7.1. Our results demonstrate that varying the composition of the preassembly buffer by reducing the Tris concentration increased filament diameters from 11.3 (22.5 mM Tris-HCl), 11.7 (21.25 mM Tris-HCl) and 12.4 nm (20.50 mM Tris-HCl). In summary, our systematic analysis revealed that a “1 mM low-Tris” buffer at pH 7.1 when added to an equal volume of pH 7.5 “Tris-buffer” produced desmin filaments that most efficiently bound CRYAB. These analyses established the experimental platform on which to optimize the binding of CRYAB to desmin filaments assembled in vitro.

Previous studies had noted that for desmin, in “5 mM low-Tris” buffer pH 7.4, the addition of chelators shifted the *s* value from 5.5 to 13 S (Wickert et al. 2005); thus, the buffer composition did alter the starting point in the assembly pathway by driving tetramer association (Lopez et al. 2016).

### Chaperone role for CRYAB in desmin assembly.

**Fig. 4:**
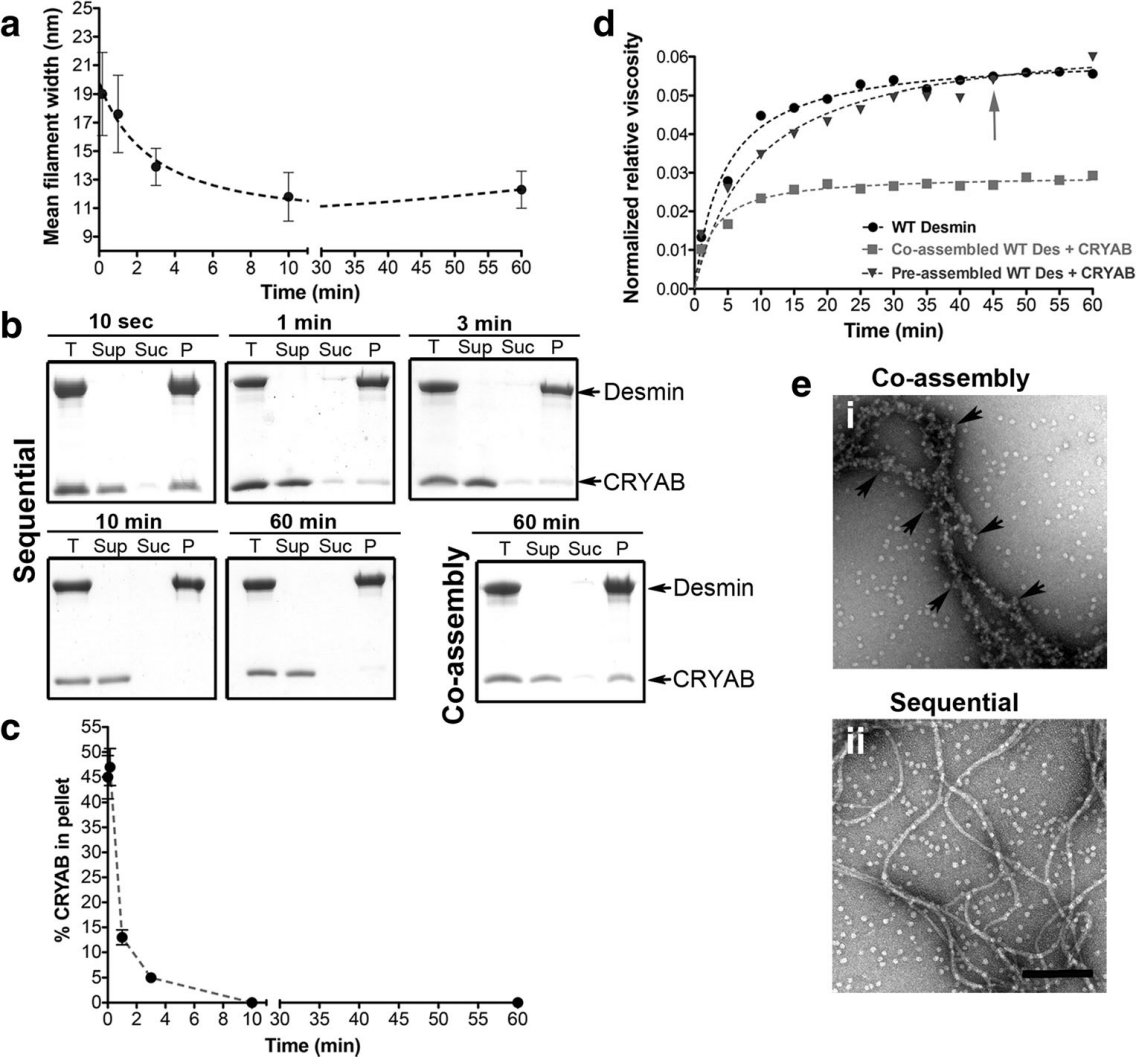
CRYAB binds to desmin assembly intermediates. **a** Graph of the change in desmin filament widths at different time points after the initiation of assembly. **b** CRYAB was added sequentially (sequential) to desmin oligomers at the indicated time points and their interaction analysed by co-sedimentation assay. For comparison, CRYAB was also coassembled with desmin (coassembly). **c** CRYAB binding to desmin sharply decreases as the filaments assemble via the sequential assembly regime, yielding undetectable CRYAB binding after 10 min. The mean values (mean ± SD) of three independent assays are shown. All samples were assembled for a total duration of 120 min. Abbreviations for collected fractions: *T* total, *Sup* supernatant, *Suc* sucrose, *P* pellet. **d** Viscometry measurements were obtained for WT desmin alone (*circle*), desmin coassembled with CRYAB (*square*) and the addition of CRYAB after 45 min (*arrow*) to a desmin assembly mix (*triangle*). Coassembly of CRYAB with desmin reduced the sample viscosity by 50% as compared to WT desmin or the preassembled desmin. **e** Electron micrographs compare desmin coassembled with CRYAB after 120 min (*i*) to CRYAB added after 60 min to preassembled desmin and incubated for a further hour (*ii*). Note that with CRYAB coassembly, the filament backbone of desmin is almost totally covered by CRYAB particles. Scale bar = 100 nm

The next step in our optimization strategy tested whether CRYAB would bind equally well to the early or late stages of desmin filament assembly. To this end, we manipulated the timing of the addition of CRYAB to the desmin assembly reaction and collected samples for EM to monitor the association of CRYAB with desmin. Simultaneously, we recorded the partition into soluble and insoluble fractions by co-sedimentation and the effect on network properties by Ostwald viscometry (Fig. 4). To characterize the stage of filament assembly, we measured the diameter of the desmin filaments over the time course of assembly. We found that the radial compaction reached a minimum diameter of 11.8 ± 1.7 nm after around 10 min of assembly, starting from 19 ± 2.9 nm after 10 s, 17.6 ± 2.7 nm after 1 min and 13.9 ± 1.3 nm after 3 min (Fig. 4a; (Herrmann and Aebi 2016). In the coassembly regime, CRYAB and desmin were mixed prior to the initiation of filament assembly, whilst in the sequential regime, CRYAB is added at different time points after the initiation of desmin assembly and stopped deliberately when the elongation and compaction phase of filament assembly occurred (Herrmann and Aebi 2016; Lopez et al. 2016). Thus, CRYAB was added at different time points during the radial compaction phase, and its coassembly with desmin filaments was compared to samples prepared using the coassembly regime, when both proteins are mixed before the initiation of assembly (Fig. 4b; coassembly). If CRYAB was added within the first 3 min, then the chaperone was found to pellet well with desmin in the co-sedimentation assay. If, however, CRYAB was added at later time points, then no association was detected (Fig. 4b, sequential, 10 and 60 min). When CRYAB was coassembled simultaneously with desmin, a stable association was detected upon assay termination after 60 min (Fig. 4b; coassembly). The levels of pelletable CRYAB were determined by quantifying CBB bands after SDS PAGE (Fig. 4c). These data indicate a time dependency in the association of CRYAB with the assembling desmin filaments.

Next, we compared the viscosity profiles of the desmin filament networks formed during the coassembly and sequential assembly regimes as described in Fig. 4a. When CRYAB was coassembled with desmin, then a 50% drop in the viscosity was observed. If CRYAB was added 45 min after the initiation of filament assembly, then no decrease in viscosity was seen (Fig. 4d). Visualization of the desmin and CRYAB by EM confirmed that by coassembly, CRYAB coated the desmin filaments (Fig. 4e (i)). In contrast, when CRYAB was added after 45 min, this association was not readily seen (Fig. 4e (ii)). These data are in close agreement with our co-sedimentation and viscosity data (Fig. 4b–d) and furthermore suggest that CRYAB is able to sense differences in the assembling desmin filaments.

### The tail domain of desmin harbours critical CRYAB-binding sites

The C-terminal domain of vimentin and desmin is recognized for its role in modulating filament width control and filament-filament interactions (Herrmann et al. 1996, Bar et al. 2007a, Bar et al. 2010). Cardiomyopathy disease-causing desmin tail mutations do not coassemble correctly with WT desmin in vitro and in transient transfection of myoblasts and have a notably decreased network viscosity, suggesting that the tail domain of desmin is important for biomechanical force transmission between myocytes (Bar et al. 2007a; Bar et al. 2010). Passive rheology measurements indicated that CRYAB likely modulates the desmin network viscosity (Elliott et al. 2013). These findings are consistent with our bulk network viscosity measurements (Fig. 4d. Therefore, based on these findings, we sought to investigate in further detail how the C-terminal domain of desmin might modulate CRYAB binding.

**Fig. 5:**
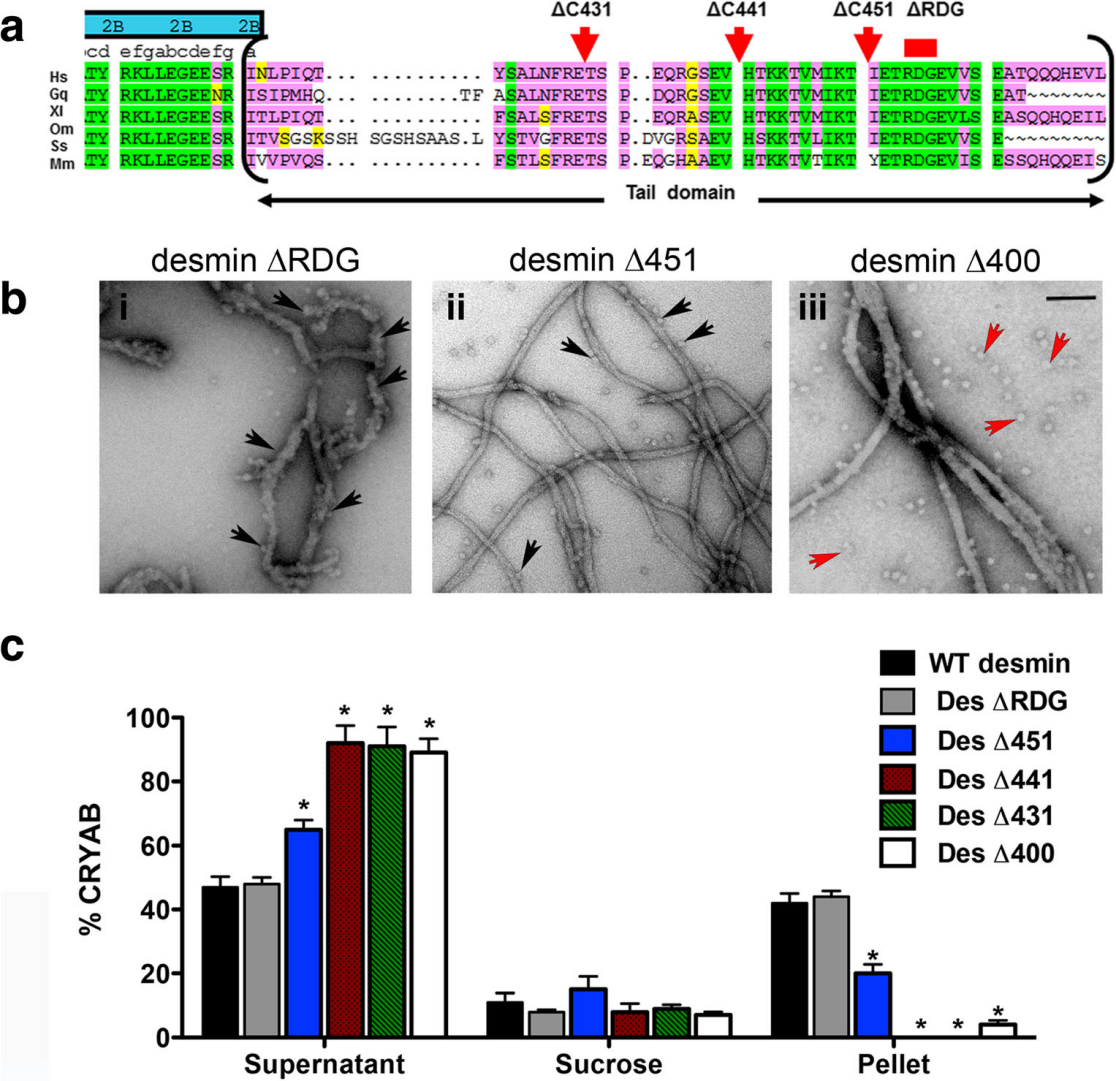
CRYAB binds to residues 452–470 in the C-terminal tail domain of desmin. Amino acid sequence comparison of the non-α-helical tail domain of desmin is shown (**a**). Six species are represented (*Hs Homo sapiens*, *Gg Gallus gallus*, *Xl Xenopus laevis*, *Om Oncorhynchus mykiss*, *Ss Scyliorhinus stellaris*, *Mm Mus musculus*). The global sequence alignment demonstrates that the RDG motif is highly conserved. **b** Electron micrographs show strong recruitment of CRYAB particles to Des ΔRGD (**b** *i*), moderate recruitment to Des Δ451 (**b** *ii*) and weak recruitment to tailless desmin Des Δ400 (**b** *iii*) filaments. Unbound CRYAB particles were observed in the sample containing tailless desmin. *Black arrows* indicate CRYAB particles bound to desmin, whilst *red arrows* indicate free CRYAB particles. Assembly was stopped at 60 min by addition of 0.1% (*v*/*v*) glutaraldehyde. Scale bar 100 nm. **c** Graph shows the band intensity quantification for co-sedimentation fractions (supernatant, sucrose and pellet) of CRYAB and five desmin tail deletions. More CRYAB binding occurred with Des ΔRDG, followed by Des Δ451. In contrast, Des Δ441, Des Δ431 and Des Δ400 showed very little CRYAB binding. *Bars* indicate the mean value of three independent experiments

For this purpose, a series of tail truncation desmin proteins were purified, and their interaction with CRYAB was systematically tested by co-sedimentation assays (Fig. 5). Alignment of desmin from multiple species shows a high degree of sequence conservation for the RDG and IKT motifs (Fig. 5a). The binding of CRYAB to Des ∆RGD was indistinguishable to that of WT desmin by examination of their ultrastructure using EM. In contrast, many unbound CRYAB oligomers were detected when imaged with tailless Des∆400 protein (Fig. 5b). Band densitometry analyses of co-sedimentation assay fractions revealed that deletion of the conserved motif RDG yielded ~44% recovery of CRYAB in the pellet fraction. This was nearly halved (~20%) by the deletion of the last C-terminal 29 amino acids of desmin (Des∆451). The interaction was completely abrogated when Des∆441 and Des∆431 were assessed for CRYAB binding (Fig. 5b). Our results indicate that CRYAB binding most likely occurs between the amino acid residues 442–453 of desmin (SEVHTKKTVMIKTIET).

### CRYAB has opposing binding defects for desminopathy-linked tail mutants

**Fig. 6:**
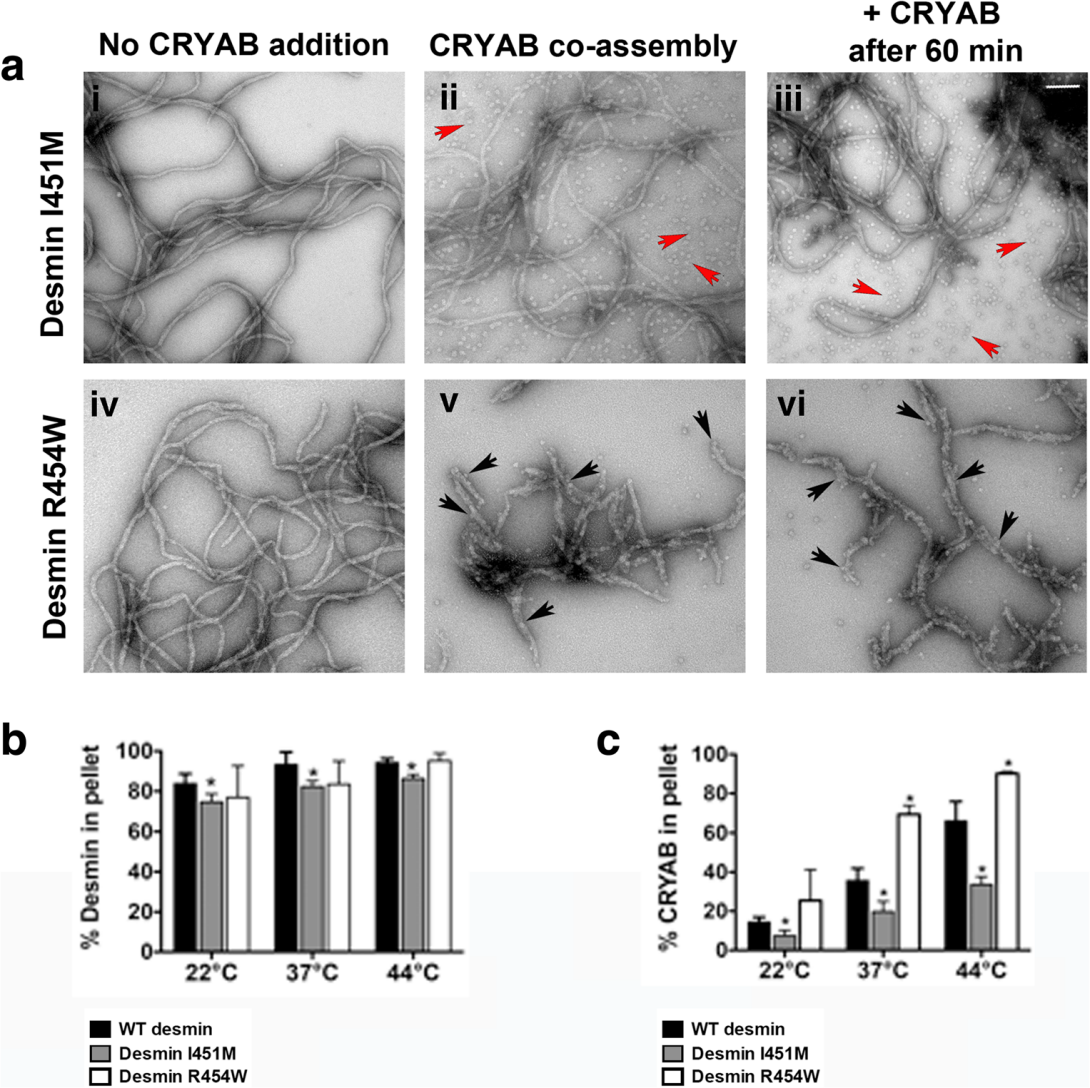
CRYAB distinguishes desmin mutants based on their filament surface topology. The mutant desmin I451M (**a** *i*) showed seemingly normal filament topology, similar to that observed for WT desmin (not shown). In contrast, mutant desmin R454W formed loose and wider filaments (**a** *d*). CRYAB binding is much lower for mutant desmin I451M under the two assembly conditions (**a** *ii*, **a** *iii*; free CRYAB particles indicated by red arrows), whereas it readily bound to mutant desmin R454W (**a** *v*, **a** *vi*; *black arrows*). Assembly reactions were stopped by adding 0.1% (*v*/*v*) glutaraldehyde prior to imaging by electron microscopy. Scale bar 100 nm. Bar graph shows the band intensity for the pellet fractions obtained after co-sedimentation analyses (**b**, **c**). Three temperatures (22, 37 and 44 °C) were used for co-sedimentation analysis after coassembly of CRYAB with desmin for 1 h, to compare the sedimentation profiles of WT desmin to mutant I451M and R454W desmin (**b**) and the fraction of co-sedimenting CRYAB (**c**). At all temperatures, the mutant Des R454W strongly bound to CRYAB, whilst mutant desmin I451M evidenced a weaker binding (**c**). The mean ± SD of at least three independent experiments is shown for each data series. Significant differences are indicated (*P < 0.05%)

Two desmin mutants located in the C-terminal tail domain that cause desminopathy (I451M and R454W) were then assessed for their CRYAB binding. Unexpectedly, we found as assessed by co-sedimentation assays that binding of CRYAB to mutant desmin resulted in altered binding as compared to desmin WT (Fig. 6). Examination of filaments of mutant desmin I451M coassembled with CRYAB showed reduced CRYAB binding (Fig. 6a (ii, iii)). In contrast, electron micrographs of mutant desmin R454W revealed enhanced CRYAB binding covering the surface of these filaments (Fig. 6a (iv, v)). An assembly kinetic study of the assembly intermediates formed by these mutant desmins showed clear structural differences on the filaments, indicating that the desmin R454W mutation blocked the radial compaction (Bar et al. 2007a). The I451M desmin mutant on the other hand was noted for its assembly into smooth filaments, but the dramatically increased viscosity of the sample (Bar et al. 2007a) is indicative of increased filament-filament interactions. In line with these findings, other structural studies predict that most of the tail domain of desmin projects outward from the core of the filament, reviewed in (Herrmann and Aebi 2016).

CRYAB plays a central role in stress tolerance in muscle cells. As such, more CRYAB binds to desmin at elevated temperatures (Elliott et al. 2013; Perng et al. 2004), and temperature elevation increases sHSP subunit exchange and potential chaperone activity (Aquilina et al. 2013; Datskevich and Gusev 2014). In this study, we compared the ability of CRYAB to function as a desmin sensor in physiological temperatures and raised temperatures (Fig. 6b, c). Both I451M and R454W desmin were not as efficient as wild-type desmin in their assembly as measured by the pelletable protein (Fig. 4b), but these differences were only significant for I451M for which 74.6, 82.3 and 86.3% protein was pelleted at 22, 37 and 44 °C, respectively (cf 83.8, 93.3 and 94.3% at 22, 37 and 44 °C, respectively, for wild-type desmin). Our EM data were consistent with the biochemical analyses as the percentages of CRYAB recovered in the pellets after in vitro assembly increased in a temperature-dependent manner (22, 37 and 44 °C, Fig. 6c). Our data show that significantly more CRYAB bound to mutant desmin R454W filaments as compared to WT desmin at 37 and 44 °C. Our data also revealed significantly lower amounts of CRYAB bound to mutant desmin I451M at all three temperatures investigated (Fig. 6c), more than could be explained by the small reduction in its sedimentation properties (halving in CRYAB levels for a 10–15% decrease in sedimentable desmin). These data indicate that CRYAB detects differences in filament surface topologies of disease-causing desmin mutants. We conclude that these findings suggest a novel sensing function for CRYAB with respect to detecting and surveying desmin filament subunit topology during its assembly.

### The R454W mutation in desmin drives the association of CRYAB

**Fig. 7:**
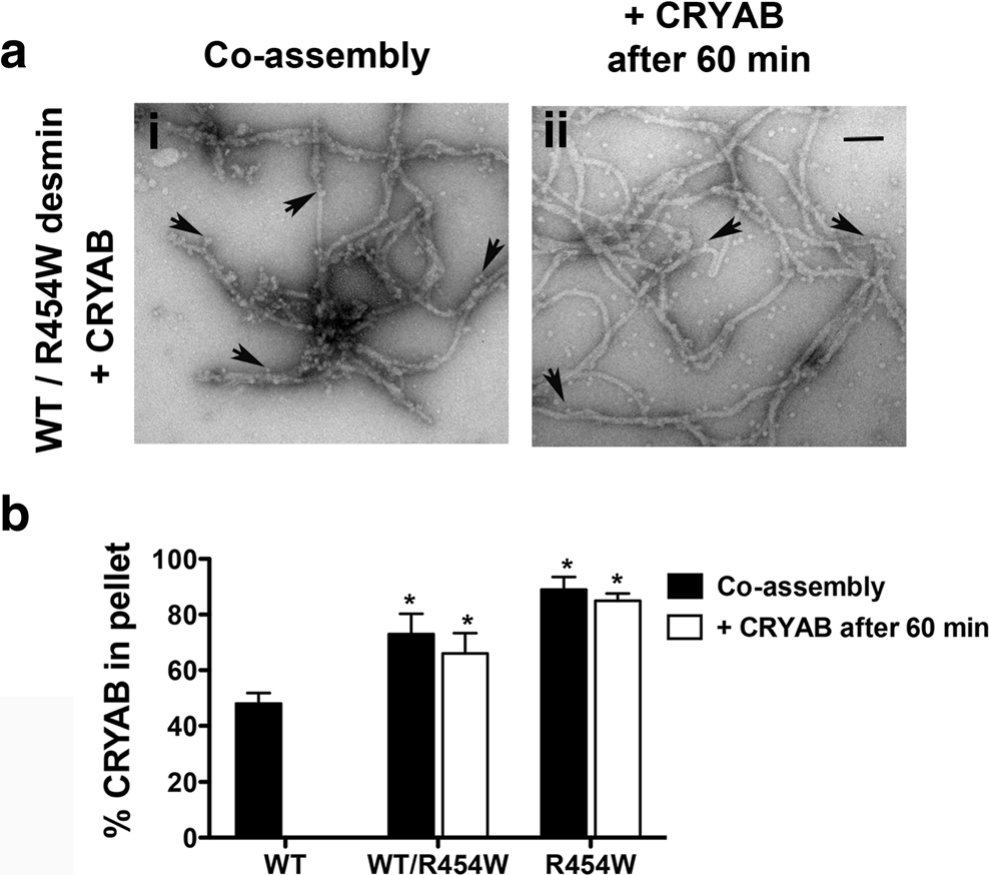
Alterations in the filament topology of mutant desmin R454W promote excessive CRYAB binding. **a** Electron micrographs compare the CRYAB bound to desmin filaments using equimolar mixtures of WT and R454W desmin in either coassembly (**a** *i*) or addition of CRYAB 60 min after assembly was started (**a** *ii*). Samples were fixed in 0.1% (*v*/*v*) glutaraldehyde after assembly prior to EM. Bound CRYAB particles are indicated (*arrows*). Scale bar 100 nm. **b** Bar graph shows the quantification of pelletable CRYAB obtained after coassembly with the different desmin preparations. When coassembled, WT desmin exhibited decreased binding to CRYAB as compared to heterozygous or homozygous mutant desmin R454W (*black bars*). No binding to WT desmin was detected when CRYAB was added after filament maturation. In contrast, both heterozygous and homozygous desmin R454W show enhanced CRYAB binding (*white bars*). The mean ± SD of three independent experiments is shown

We used EM (Fig. 7a) and co-sedimentation (Fig. 7b) assays to determine the impact of the desmin R454W mutant on the CRYAB binding to WT desmin. EM analyses comparing the filament morphology of heterozygous to homozygous desmin on coassembled or preassembled desmin prior to the addition of CRYAB revealed little difference at this resolution. In both cases, CRYAB bound along the entire filament length (Fig. 7a). The desmin R454W mutant was mixed in equal ratio with WT desmin to mimic of the autosomal dominant impact of this mutation (Clemen et al. 2005). When CRYAB was added to WT desmin 45 min after the initiation of assembly, no binding was seen (Fig. 5). In contrast, when WT desmin was coassembled with R454W, then ~66% CRYAB was recovered in the pellet fraction. In fact, the amounts of CRYAB recovered in the pellet were similar to mutant desmin alone (Fig. 7b). Specifically ~73% and ~89% CRYAB was recovered in the pellet fractions of samples prepared by the sequential and coassembly regimes. We conclude that the assembled filaments are topologically altered by the incorporation of R454W desmin, encouraging CRYAB binding.

## Discussion

In this study, we report that desmin filament morphology influences CRYAB binding suggesting that it can act as a sensor detecting changes in the surface topology of IFs. It is well established that the assembly protocol, i.e. rapid versus dialysis (Herrmann et al. 1999) as well as the monovalent or divalent cation concentration (Brennich et al. 2014) all influence the diameter of the desmin filaments produced (Stromer et al. 1987). In the case of vimentin, a structurally related IF protein to desmin, the MPL values of filaments formed by dialysis exhibited a homogeneous filament population rather than the polymorphism seen by rapid assembly protocols (Herrmann et al. 1996). We suggest this indicates desmin subunit topology is an important aspect of filament structure and therefore its function. CRYAB binding is dramatically influenced by desmin filament morphology (Figs. 1 and 2). Here, we deliberately used two in vitro assembly protocols to produce assembled filaments of different diameter, in order to manipulate the mass-per-length (MPL; (Herrmann et al. 1999)), and the data we have presented here suggest that the resulting topological differences are detected by differences in the extent of CRYAB binding and forming a stable complex with the IFs.

The prevalent model for IF assembly is largely based on length measurements of vimentin IFs at different time points (reviewed in (Herrmann and Aebi 2016), since vimentin and desmin belong to the same assembly group and can co-polymerize (Quinlan and Franke 1982) and therefore share a common assembly pathway (Herrmann and Aebi 2016). Filament assembly begins as tetramers associate laterally to form ULFs (phase 1), next ULFs anneal longitudinally into filaments (phase 2) and then filaments undergo a radial compaction event (phase 3; (Herrmann and Aebi 2016)). This process occurs very fast, since during the first 10 s of assembly, ~85% of all subunits are assembled into ULFs, and subsequently ULFs are consumed within the 10 min of assembly (Lopez et al. 2016; Sokolova et al. 2006). In the assembly regimes utilized here, we show that CRYAB rapidly binds desmin subunits within the first 3 min of assembly (Fig. 4b, c). This finding is consistent with the ideas that chaperones can modulate IF assembly (Landsbury et al. 2010; Nicholl and Quinlan 1994; Perng and Quinlan 2015). Accordingly, it has been demonstrated that HSP27 (HSPB1) regulates the early assembly and bundling dynamics of keratin K8/K18 IF networks (Kayser et al. 2013), with the well-documented role of protein chaperones in assisting protein assemblies (Ellis 2013). We also demonstrate that the C-terminal domain of desmin is important for CRYAB binding, consistent with previous reports that implicate this domain of desmin in high-order filament organization (Bar et al. 2007a; Bar et al. 2010).

Once the compaction stage has started during the in vitro assembly of desmin, then little CRYAB binding will occur (Fig. 4b, c). If, however, desmin filaments are prepared by serial dialysis, CRYAB can still bind (Nicholl and Quinlan 1994) arguing that CRYAB-binding sites remain accessible in some assembly regimes, but not in others as we evidence here (Figs. 1 and 2). The inclusion of CRYAB at the start of the assembly process ensures that the desmin filaments retain their CRYAB binding, reducing their solution viscosity (Fig. 4d; (Elliott et al. 2013)). Such effects are lost if CRYAB is added once filament compaction has occurred (Fig. 4b, c, e (ii)). We suggest that even though morphologically there appears to be little difference, CRYAB can distinguish the stage of desmin assembly and filament end products. Our data are consistent with the kinetic trapping mechanisms reported for reconstituted actin and keratin cytoskeletal networks (Kayser et al. 2013). Such regulation is a recognized feature of other self-assembly systems (Yan et al. 2016) from viral capsids (Cardone et al. 2014) to individual proteins (Barducci et al. 2013) and suits the emerging role for sHSPs as sensors (McHaourab et al. 2009) capable of using the energy landscape of protein assembly and particularly IFs.

### The C-terminal domain of desmin is required for CRYAB binding

Our optimization strategy investigated the effects of pH and ionic strength using Tris buffers at different molarities (Fig. 3b). We used these to show that the C-terminal tail domain of desmin was important for CRYAB binding. CRYAB also detects changes to filament topology as result of the incorporation of desmin with mutations in the C-terminal tail domain (Figs. 5, 6 and 7). This domain regulates desmin filament bundling (Kaufmann et al. 1985) as corroborated by the unique viscometric profiles of the truncated proteins (Bar et al. 2007a; Bar et al. 2010; Herrmann et al. 1996). The complete removal of the C-terminal domain does not alter the in vitro assembly of desmin (Rogers et al. 1995), but it could influence the subunit conformation in the filaments as well as affecting the availability of docking sites for IF-associated proteins (Herrmann and Aebi 2016; Rogers et al. 1995). In transfected cells, the C-terminal tail of desmin affects the higher order organization of filaments (Bar et al. 2007a; Herrmann et al. 2003). Disease-associated tail mutations in desmin change the CRYAB-binding properties (Figs. 6 and 7) and also help explain the previously described altered filament morphology (R454W) and aberrations in filament bundling (I451R) desmin structures (Bar et al. 2010). The dominant nature of the R454W mutation in CRYAB binding to assembled filaments was particularly striking and resonates with the histopathological feature of desminopathies. Together, our data suggest that the surface topology of desmin filaments strongly influences the binding of CRYAB, expanding the role of this chaperone from merely modulating filament assembly to also sensing changes in the filament surface topology.

### Role of CRYAB in desminopathy

A hallmark of biopsied muscles in desminopathy is the presence of granulofilamentous aggregates (Clemen et al. 2013; van Spaendonck-Zwarts et al. 2011). Typically, the accumulation of IFs, chaperones and sarcomere proteins in insoluble amorphous protein aggregates disrupts the alignment of serially connected Z-discs and the extra-sarcomeric linkages of muscle myofibers to the intercalated discs (Maerkens et al. 2013). Desminopathy caused by R454W desmin mutation is associated with disintegrated Z-discs and severe misalignment of myofibrils (Bar et al. 2007a; Mavroidis et al. 2008). One major role of CRYAB is to prevent the incorrect self-association of desmin filaments that may produce aggregates in cells (Houck et al. 2011), given that in vitro, the CRYAB alleviates desmin aggregation (Elliott et al. 2013).

Overexpression studies of mutant desmin I451M and R454W in C2C12 myoblasts show that both mutants can be readily incorporated into the endogenous IF networks; however, in humans, they cause severe cardiomyopathy and skeletal muscle disease (Bar et al. 2007b). Structurally, it is known that desmin R454W produced unravelled open filamentous structures, whilst mixtures of mutant desmin I451M and WT desmin yielded abnormal bundles (Bar et al. 2007a). These previous observations are consistent with our findings that both of these mutant desmin exhibited abnormal filament topology, and this is reflected in the altered CRYAB binding reported here (Figs. 6 and 7). We interpret this as evidence for the ability of CRYAB to detect changes to the surface topology of the in vitro assembled desmin filaments as a result of the incorporation of these mutant desmins.

### The assembly chaperone and sensor properties of CRYAB toward desmin

**Fig. 8:**
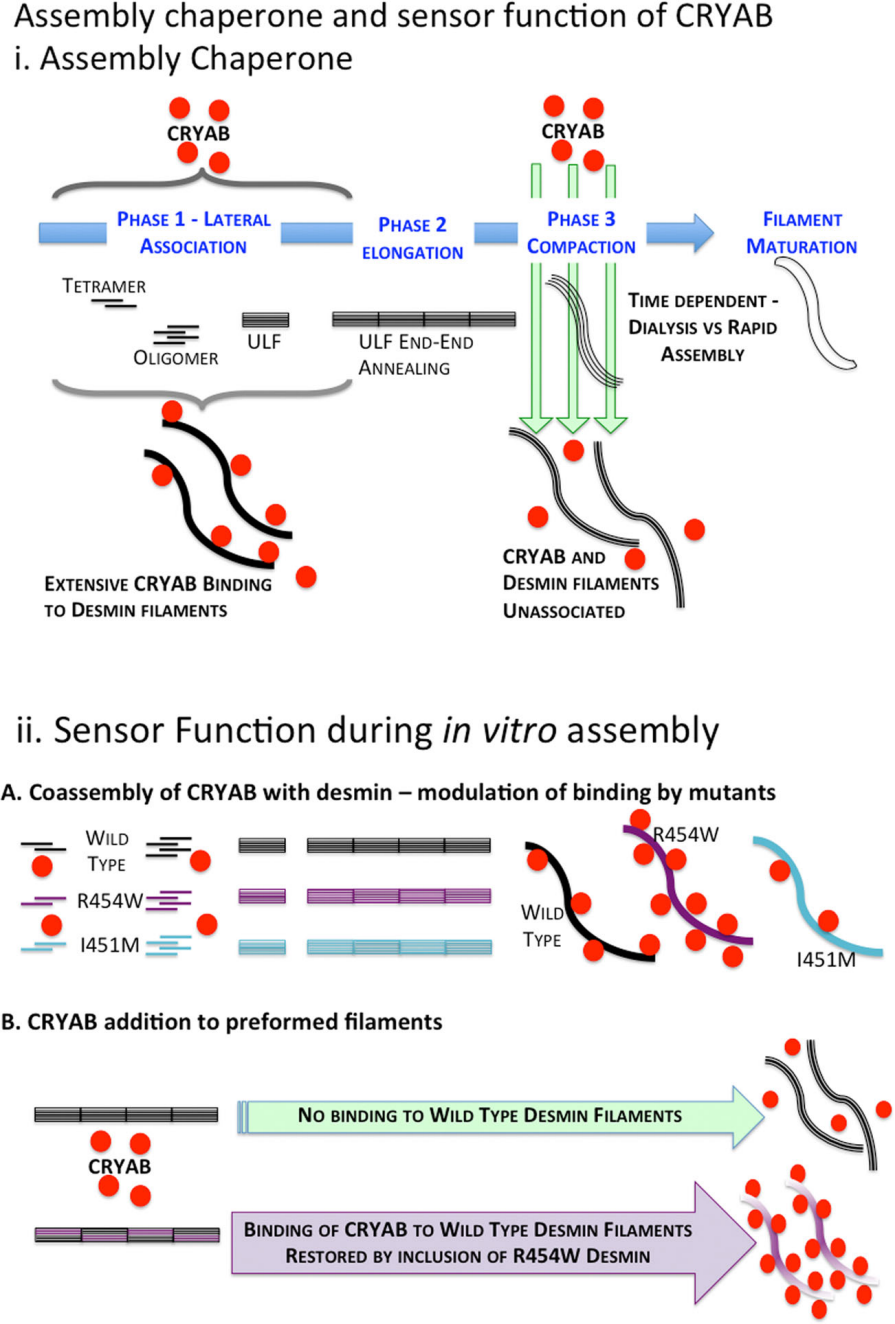
CRYAB senses desmin filament morphologies and also performs an assembly chaperone role. **a** When CRYAB is coassembled with desmin, it binds rapidly during the lateral association and early elongation phases (phases 1 and 2) of desmin filament assembly, but there is reduced binding in the later stages when compaction of the assembled ULFs occur. Note that when coassembled with CRYAB, the desmin filaments retain their CRYAB-binding properties; it is only once compaction is reached that CRYAB binding is prevented when the rapid assembly protocol is used. This exemplifies the chaperone assembly property of CRYAB with respect to desmin filaments, an association that then determines the subsequent network properties as seen by the reduced viscometric characteristics. The rapid assembly protocol utilized here differs to the dialysis regime used in previous studies (Elliott et al. 2013; Nicholl and Quinlan 1994). **b** The binding of CRYAB to WT, I451M and R454W desmin illustrates the sensor potential for CRYAB with respect to assembled desmin filaments. Mutant desmin R454W bound significantly more, whilst mutant desmin I451M bound less than WT, despite the fact that filament diameter and the gross morphological features of the filaments were similar. Moreover, the coassembly of mutant desmin R454W and WT desmin re-established CRYAB binding to compacted filaments. Once again, CRYAB detects filaments with altered surface properties, which revealed no gross morphological changes. CRYAB is therefore a biosensor for the surface topology of desmin filaments

We propose as a working model that desmin filaments utilize the binding of CRYAB during its assembly process to influence the topology of the filaments formed and potentially also modulate the temporal interaction to other binding partners in the sarcomere (Fig. 8). Here, we evaluated CRYAB binding to desmin filaments with different surface topologies. Our findings support the idea that assembly chaperone function of protein chaperones (Ellis 2013) is valid specifically with regard to IF assembly and its interaction with CRYAB (Landsbury et al. 2010; Perng and Quinlan 2015). A key finding of this study was that mutant desmin linked to desminopathy changed the desmin filament topology, and that it significantly altered its binding affinity to CRYAB. These data strongly indicate that CRYAB recognized the mutant desmin filament surface as abnormal. Interestingly, nebulin, an integral protein of the sarcomere that binds desmin, also recognizes differences in mutant desmin filaments surface topology in mutations mapping to the head and the coil 1B domains of desmin (e.g., desmin S46F and E245D, respectively) (Baker et al. 2013).

Although the precise structural details for filament surface topology sensor function for CRYAB or nebulin are as yet undetermined, our data support a model in which the degree of CRYAB interaction with desmin varies in response to the filament surface dissimilarities (Fig. 8). In other words, CRYAB is a sensor for intermediate filament topology. At the same time, the filament surface topology can be a consequence of the involvement of CRYAB in the assembly pathway, linking the assembly chaperone and sensor aspects of CRYAB with respect to IFs. In cells and tissues, the formation of mechanically active IF cytoskeletal networks is highly regulated (Capetanaki et al. 2015). Thus, alterations in the binding of CRYAB to mutant desmin lead to either building highly dense or loosely compacted IF networks that will also impact CRYAB function in IF assembly and in muscle itself (Diokmetzidou et al. 2016).

IFs are dynamic structures both in vitro and in vivo (Herrmann and Aebi 2016). In support of our model, it was reported that muscle contraction drives the association of CRYAB to desmin-rich compartments in myocytes (Frankenberg et al. 2014), and that the concomitant pH reduction that accompanies contraction favours CRYAB association to desmin filaments (Elliott et al. 2013). In cells, IF subunits exchange along the length of the filament, modulated by kinases, phosphatases, ATP levels and other post-translational modifiers (Chang et al. 2009; Colakoglu and Brown 2009; Vikstrom et al. 1991). In vitro experiments, as seen ex vivo experiments filaments can fragment and anneal end-to-end (Noding et al. 2014; Winheim et al. 2011). Therefore, a changing surface filament topology is a novel feature of IF structure and function and an aspect of their physiology that clearly involves CRYAB and other sHSPs (Landsbury et al. 2010; Perng and Quinlan 2015) that remains to be further examined in the context of beating myocytes.

## Abbreviations

CBB: Coomassie Brilliant Blue G-250
CRYAB: AlphaB-crystallin
EM: Electron microscopy
IF: Intermediate filament
MPL: Mass-per-length
SD: Standard deviation
ULF: Unit-length filament
WT: Wild-type
